# TKA with retained hardware guided by intraoperative ultrasonography – a case report

**DOI:** 10.1186/s12893-019-0585-6

**Published:** 2019-09-02

**Authors:** Tomasz Poboży, Konarski Wojciech, Martyna Hordowicz

**Affiliations:** 1 Department of Orthopaedic Surgery, Medicover Hospital, Warsaw, Poland; 2 Department of Orthopaedic Surgery, Ciechanów Hospital, Ciechanów, Poland; 3 Hospice of St. Christopher, Outpatient Pain Clinic, Warsaw, Poland; 4 GSK Poland, Medical Advisor & MSL in Dermatology & Established Portfolio, Warsaw, Poland

**Keywords:** TKA, Extramedullar, Retained hardware, Gonarthrosis, Ultrasonography

## Abstract

**Background:**

When a conservative management of gonarthrosis yields unsatisfactory results, a total knee arthroplasty (TKA) is recommended as an alternative approach. The implant survival is crucial for the long-term success of the procedure. However, in case of patients with retained hardware after past orthopedic procedures, providing correct alignment of the implant, which contributes to its longevity, is especially challenging. Here we present the use of an intra-operative ultrasonography for implant positioning in a 83-year-old male, undergoing TKA, without hardware removal, which was contraindicated due to his advanced age and comorbidities. Other imaging modalities taken into consideration are also described.

**Case presentation:**

The right knee joint was approached with anterior incision. A femoral guide was introduced extramedullary. Ultrasonography was used to pinpoint the center of the femur’s head. Tibial’s guide was introduced intramedullary followed by a standard cut of the proximal part. Cemented ZIMMER NEXGEN prosthesis was used. Layered closure was applied. The placement of implant in neutral axis was confirmed on radiographs.

**Conclusions:**

Our case demonstrates that ultrasonography might be helpful during TKA-procedure for implant positioning. However, more studies are needed to evaluate accuracy and application of ultrasound in the intraoperative settings.

## Background

With increasing life expectancy incidence of osteoarthrosis rises – which is observed in developed, countries [1]. According to the American Academy of Orthopedic Surgeons and American College of Rheumatology guidelines, in initial management of gonarthrosis, various non-pharmacological (such as exercise, weight-loss and physiotherapy) and pharmacological interventions (including both nonsteroidal anti-inflammatory drugs and opioids) are used for symptom management [2, 3]. However, when conservative management of gonarthrosis does not provide sufficient pain relief - surgical interventions, such as arthroscopy, and joint replacement - total knee arthroplasty (TKA) - remains an alternative. In Poland more than 20.000 TKA procedures are performed each year. Success of the procedure relies on time of implant survival, for which correct alignment of implant is considered the most important factor. In order to improve the accuracy of implant’s components alignment, intra-medullar (IM) guides are routinely used [2]. However, in some circumstances – for instance in patients with retained hardware after previous surgery - their use might not be possible.

Pre-operative presence of retained hardware in the ipsilateral lower limb makes TKA particularly challenging. History of surgical interventions may influence local anatomical conditions, making placement of prosthetic material along the mechanical axis particularly difficult. Removal of hardware is usually advised, but it is linked with higher risk of complications such as periprosthetic fractures and poorer outcome [4]. Therefore, in presence of retained hardware, usage of various technical aids such as computer-assisted surgeries (CAS) and patient-specific blocks (PSP) have been described the most frequently [5]. As for today there were no cases of intra-operative ultrasonography use for the assessment of implant’s elements positioning described in the literature.

## Case report

An 83 year-old man presented with a pain in the right knee of 8 in VAS (visual analogue scale). The symptoms were worsening over the last few years. He had a history of arterial hypertension, glaucoma and cataract. Forty years ago he had right femur fracture, and underwent surgical intervention. Plate and screws after osteosynthesis were not removed, and no documentation on the details of the intervention were available. He also underwent prostatectomy, appendectomy and hemorrhoidectomy in the past. The patient signed an informed consent for publishing his case.

**Fig. 1:**
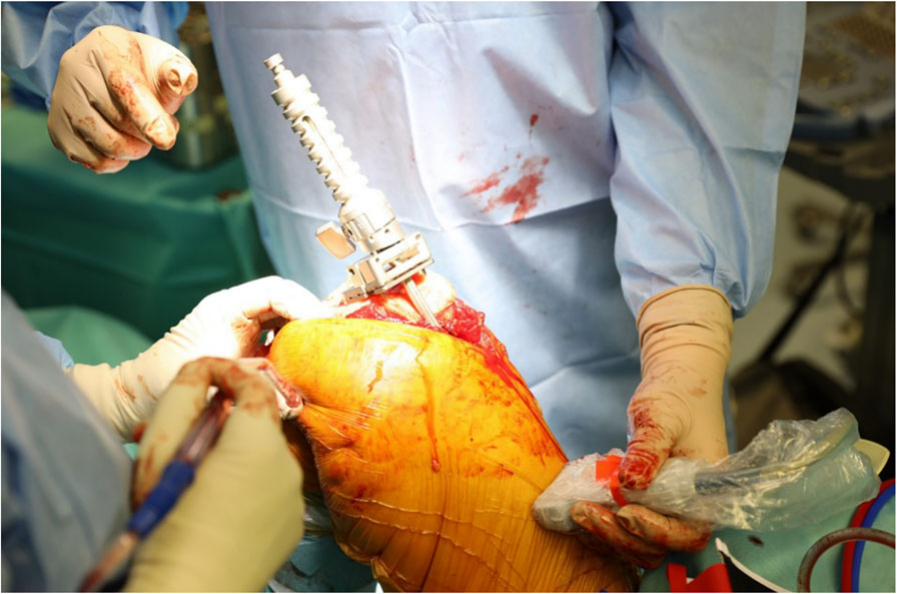
Ultrasound head placement to confirm extramedullary guide positioning

On admission the patient was stable, BP 125/85 mmHg, HR 72/min. Initial laboratory results were within normal values. Pre-procedurally the patient was administered ananticoagulant according to local standards (enoxaparin, 40 mg) and a prophylactic dose of antibiotic (cefazoline) along with his regular drug regime. Based on the radiological features of right lower limb joints the patient was qualified for total knee arthroplasty with a cemented knee prosthesis - NexGen (LPS-Flex) implant (Fig. 1).

**Fig. 2:**
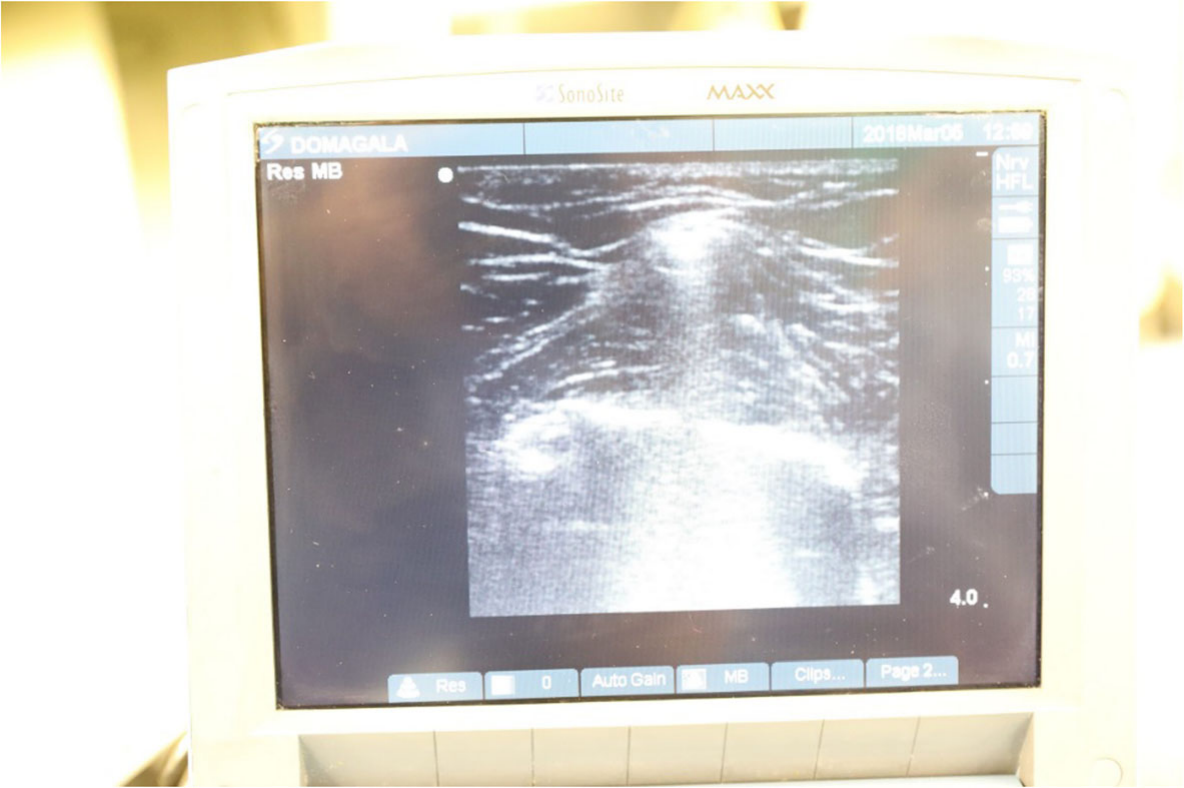
Ultrasound image demonstrating placement of the guide along femoral shaft and bone

Due to long time interval between past surgery and current intervention and concomitant diseases, the patient was not found eligible for hardware removal. Extensive surgery including simultaneous hardware removal and TKA could put the patient at unacceptably high risk of complications (such as infection, perioperative fracture, and significant blood loss), because of his advanced age and comorbidities (Fig. 2).

Placement of femoral guide intramedullary (which is preferred by most surgeons because, as it makes fitting of the prosthetic material easier, because the rod goes along anatomical axis of the limb) was not possible due to the presence of retained hardware. Its presence would not allow passage of intramedullary rod. Therefore, a decision to use an extramedullary guide was made preoperatively. Correct insertion of extramedullary guide is found challenging, because visual assessment of reference points used to determine the correct positioning is difficult intraoperatively. However, because confirmation of guide’s correct position is vital in order to achieve a good long-term outcome, ultrasound was chosen to inspect its position along the mechanical axis, being an accessible, inexpensive and a non-invasive imaging modality that could be performed intraoperatively by a trained member of the surgical team.

Several other imaging methods were considered. Fluoroscopy was not found to be optimal, because it requires a specific position of the limb (flexion in the hip and knee joint (of 90 degrees). It is also linked with an exposure to radiation, which forms its disadvantage in comparison with ultrasound. Using a patient specific instrument (PSI) was also not applicable to this case. It imposes performing an MRI of the limb, which was contraindicated. There was no available documentation of the previous surgery, therefore the metal alloy of the retained hardware was unknown.

TKA was performed in supine position. The right knee joint was approached with anterior incision. Numerous degenerative changes were present in both medial and lateral compartment, dominating in the medial compartment. Anterior compartment presented normally. Hardware retained after previous intervention, in the form of screws and an ostheosyntesis plate were present. After examining local conditions, a femoral guide was introduced extramedullary. Ultrasonography was used to pinpoint the center of the femur’s head. Distal cut in femur was performed. Tibial guide was than introduced intramedullary followed by a standard cut of the proximal part. The initial fit of implants was assessed. The patella’s osteophytes were removed. It was followed by ZIMMER NEXGEN prosthesis embedment on cement (vacuum mixed). Size of the tibial part was 5, femoral part F and polyethylene insertion – 9 mm. Layered closure was applied with the introduction of ATS reciprocal drainageto reduce oedema at the surgical site.

On the 2nd day after surgery the drainage was removed (it collected 200 ml of bloody excretion, which was within normal volumes expected after TKA). The postoperative period was uneventful. The correct position of the implants along the mechanical axis was confirmed on X-ray. On the 4th postoperative day the patient was able to walk using crutches and he was discharged from the hospital and he was advised to continue rehabilitation, use of analgesics (paracetamol 3x500mg, dexketoprofen 2x25mg taken as needed) and anticoagulant (enoxaparin 40 mg) administered daily in the first 6 weeks after discharge.

**Fig. 3:**
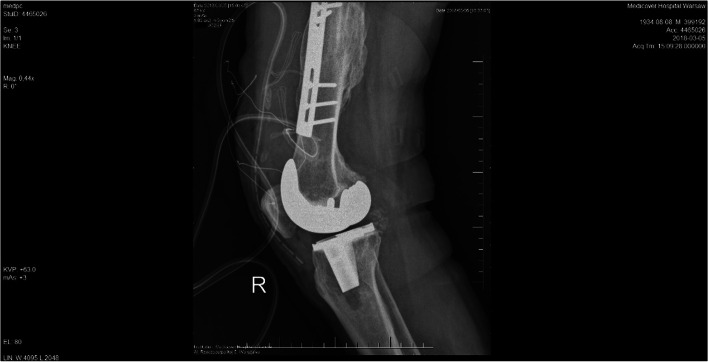
Postoperative X-ray confirming prosthesis placement in the neutral axis

At a follow-up visit 6 months after surgery the patient was generally satisfied with the results of TKA, The patient recovered full range of motion (in comparison with the contralateral side). Pain decreased significantly to 1–2 in VAS (before surgery – up to 8 in VAS). On physical examination knee joint had normal appearance, with no signs of edema (Fig. 3).

## Discussion and conclusions

The most important factor to achieve patient’s satisfaction with TKA procedure for end-stage gonarthrosis is long-term quality of life improvement and pain reduction, which can be translated into correct placement of implant – within the neutral axis. However in only around 75% of TKA’s an ideal alignment is acheived [6].

Introduction of guides for implant’s positioning can be performed intramedullary or extramedullary. Intramedullary approach is associated with better stability and durability of implants, but it is also related with a higher risk of blood loss during procedure and post-operative complications (hypoxia, fracture, fat embolism, death) [7, 8]. However, intramedullary approach is not always feasible, as in the case described above.

In our experience, the greatest challenge for a surgeon performing TKA is the presence of retained hardware from previous lower limb procedures. It is a priori connected with an increase in the risk of postoperative complications [4]. Options that can be considered before performing TKA in such patients are (1) removing the hardware, (2) implementing computer-assisted surgery techniques or (3) using an extramedullary guide. (1) Removal of retained hardware enables use of intramedullary approach, but the surgery duration is longer and it bears the highest rate of side effects – up to 28% [9]. (2) Recently published paper confirms that computer-guided arthroplasty results in lower rate of complications, but long-term observational data does not prove its superiority over traditional technique [10, 11]. Therefore in this case, an extramedullary guide assisted by ultrasound was used to confirm implant’s correct positioning.

Imaging is a necessary step before the procedure, but it may also be used intra-procedurally to localize anatomic reference points to allow correct placement of the extramedullary guide. X-ray, computed tomography and magnetic resonance imaging are usually methods of choice. Matsuda et al. (2004) states that ultrasonography indicates center of the femoral head within 5 mm in 56% cases and within 10 mm in 89,5% cases. It appears to be highly reliable, noninvasive and easily accessible imaging modality [12]. Our case describes how ultrasonography might be implemented intraoperatively to assess implant’s placement during TKA-procedure in some settings, as in the case described above - a patient with unknown details of past interventions (such as material from which the hardware was made) and concomitant diseases, which were preventing surgical hardware removal before arthroplasty. However, its use might be limited because this requires presence of a member in the surgical team who is trained in its use. A randomized controlled trial assessing intraoperative use of ultrasound in TKA procedure could further evaluate the applications and accuracy of this imaging modality.

## Data Availability

The data that support the findings of this study are available from the corresponding author, W.K, upon reasonable request. The data are not publicly available, as it might compromise the confidentiality of patient whose case was described in the case report.

## Abbreviations

ATS: Autotransfusion system
CAS: Computer-assisted surgery
IM: Intra-medullar
PSI: Patient specific instrument
PSP: Patient-specific blocks
TKA: Total knee arthroplasty
VAS: Visual analogue scale
